# The Challenge of Peat Substitution in Organic Seedling Production: Optimization of Growing Media Formulation through Mixture Design and Response Surface Analysis

**DOI:** 10.1371/journal.pone.0128600

**Published:** 2015-06-12

**Authors:** Francesco Giovanni Ceglie, Maria Angeles Bustamante, Mouna Ben Amara, Fabio Tittarelli

**Affiliations:** 1 Department of Organic Agriculture, Mediterranean Agronomic Institute of Bari, Centre International de Hautes Etudes Agronomiques Méditerranéennes, Valenzano, Bari, Italy; 2 Department of Agrochemistry and Environment, Miguel Hernandez University, Orihuela, Alicante, Spain; 3 Research Centre of soil-plant system, Consiglio per la Ricerca e la sperimentazione in Agricoltura, Rome, Italy; NERC Centre for Ecology & Hydrology, UNITED KINGDOM

## Abstract

Peat replacement is an increasing demand in containerized and transplant production, due to the environmental constraints associated to peat use. However, despite the wide information concerning the use of alternative materials as substrates, it is very complex to establish the best materials and mixtures. This work evaluates the use of mixture design and surface response methodology in a peat substitution experiment using two alternative materials (green compost and palm fibre trunk waste) for transplant production of tomato (*Lycopersicon esculentum* Mill.); melon, (*Cucumis melo* L.); and lettuce (*Lactuca sativa* L.) in organic farming conditions. In general, the substrates showed suitable properties for their use in seedling production, showing the best plant response the mixture of 20% green compost, 39% palm fibre and 31% peat. The mixture design and applied response surface methodology has shown to be an useful approach to optimize substrate formulations in peat substitution experiments to standardize plant responses.

## Introduction

Peat is the main component of seedlings growing media in the EU [1] and its use is also allowed for transplant production in organic nurseries (Regulations (EC) No 834/2007 and No 889/2008). Thus, peat-based substrates constitute the standard media used in conventional and organic seedling production. However, in recent years, the concern about the environmental impact associated with peat extraction (destruction of ecosystems highly fragile [2], potential source of C emissions [3]) has increased together with the demand of peat-based growing media in the horticultural and ornamental sectors. Furthermore, peatlands are under the safeguard of the Directive 92/43/EC for natural habitats and wild fauna and flora. Therefore, peat is considered a non-renewable resource and thus, its use must be progressively reduced. In this sense, several governments are trying to reduce the use of peat as a substrate and as a soil improver, as well as encouraging the re-use of organic wastes as substrate components instead of their disposal [4]. Nevertheless, even if the use of peat-based substrates is in contradiction with most of the basic principles of organic farming, peat is allowed in organic transplant production. This issue is perceived as very controversial and has determined an increasing need of alternative high quality and low cost materials for the partial or complete substitution of peat in the growing media. Thus, during the last ten years, an extensive research has been carried out regarding the use of different farm, industrial and consumer waste by-products as components of nursery substrates [5]. Different residual biomasses, such as coir [coconut (*Cocos nucifera* L.) husk fibre] [6], rice (*Oryza sativa* L.) hulls [7], switchgrass (*Panicum virgatum* L.) [8], spent mushroom compost (*Agaricus bisporus* (J.E. Lange) Imbach, and *Pleurotus ostreatus* (Jacq.) P.Kumm) [9], beached Posidonia residues (*Posidonia oceanica* L.) [10], extracted sweet corn tassel (*Zea mays* L.) [11], and giant reed (*Arundo donax* L.) wastes [12] have been studied as partial or total substrate components. Also, numerous studies have reported the use of organic residues, after proper composting, as peat substitutes in potting media, such as municipal solid waste compost [13, 14, 15], animal manure compost [16], green waste compost [16, 17, 18], Posidonia compost [19, 20] and agro-industrial compost [17, 21, 22, 23]. However, despite the impressive amount of data concerning the use of alternative materials, especially composts, as peat substitutes in growing media, the results concerning the suitability of these materials vary significantly and are not always satisfactory. Plant response to different substrates is strictly related to the tested species and also depends on the materials used and on the proportions in the mixtures. Not all the materials are ideal substrates for plant growth in pot, since these materials can also show limiting aspects, such as the presence of hazardous components (e.g. heavy metals), organic phytotoxins, poor physical properties, high pH and/or high salinity [18]. In this scenario, it is very complex to establish the most suitable materials and especially, the best proportions to obtain good results concerning plant growth and productivity. This fact generates a large gap between the research results obtained regarding peat substitution in growing media, which presents successful results using a peat substitution rate ranged from 40 to 80%, and the substrates available on the market, with a mean content in peat ranged between 80–100% [24].

In the studies of peat substitution with alternative materials, the experimental design is mainly limited to a specific number of mixtures (treatments), in the wide space of the opportunities that ranges from 0% to 100% of each substrate component, usually considering a linear rate of substitution (e.g. 0%, 25%, 50%, 75% and 100%) and using one or two ingredients together with peat as diluent material. In this context, at the end of any set of experiments about the effects of different substrates on transplants development, usually it is very difficult to individuate the best growing media among the others, since in most occasions the best option will be one among the tested ones. Therefore, it is necessary to establish a standard procedure that makes easier the final choice, especially for farmers and technicians, because the answer to this issue strongly affects the transplantation success. Several studies have been carried out to evaluate the impact of alternative substrates on the growth of different plant species and to assess the suitability of these materials as substrate components, using descriptive analysis and univariate inferential statistics [25, 26] or multivariate analysis [17, 18].

In this sense, the design of experiments with mixtures and the applied response surface methodology constitutes a different approach to optimize the substrate formulations for obtaining the best results regarding plant growth and productivity. In a mixture experiment design, a measured property of the mixture changes when the proportions of the components of the mixture are changed; thus, the synergetic effect of a combination of two or more components on a property of interest can be easily identified. Therefore, this innovative approach could be very useful in peat substitution studies, since this methodology can consider the specific aspects of the mixtures of heterogeneous components blended at different relative rate, which directly influence the physical, chemical and biological substrate properties, but not always as a linear function of the proportion of the mixture components. This methodology has been widely used in the optimization of formulations of food, paint, polymers, asphalt, concrete, glass and ceramic products [27], as well as in compost elaboration [28]. However, currently very little information is available regarding the use of the response surface methodology for the design of experiments related to growing media formulations. Only Moldes et al. [13] used this methodology to evaluate substrates derived from municipal solid waste compost as plant growing media components, obtaining promising results.

Therefore, the aim of this work was to evaluate and validate the use of the mixture design and the surface response methodology in a peat substitution experiment using two alternative materials (green compost and palm fibre trunk waste) for the transplant production of three vegetable species (tomato, melon and lettuce) in organic farming conditions.

## Materials and Methods

### Substrate components

The growing media of this study were elaborated using, as organic peat substitutes, green compost (GC) and palm fibre trunk wastes (PF), both coming from the Mediterranean Agronomic Institute of Bari (IAMB-CIHEAM), placed in Valenzano, (Bari, Italy; 41°03’16”N, 16°52’45”E, elevation 72 m a.s.l.). Green compost (GC) was produced at the composting facility of the IAMB-CIHEAM by using only latest pruning materials from olive trees (*Olea europaea* L.), conifer species (*Pinus* sp. and *Picea* sp.) and vegetable residues (grass (*Lolium perenne* L.) clippings) from the experimental farm of the same research centre. The mixture was managed as a trapezoidal windrow (about 2.0 x 6.0 m base, 1.5 m high) in a warehouse and was mechanically turned every day for the first week of composting and twice a week during the rest of the thermophilic phase. The moisture of the biomass under composting was monitored weekly (gravimetric weight loss). Whenever necessary, water was added to keep the composting biomass moisture at 55–60% that is the optimal range for the microbial metabolism [29]. Additional additives and/or fertilizers were not incorporated to the compost. Mature compost was sieved to 10 mm prior to be used as substrate component. Palm (*Phoenix* sp. and *Washingtonia* sp.) fibre trunk wastes (PF) were collected from a yearly stored heap of the crushed material, produced after garden maintenance activities at the IAMB-CIHEAM, and finally sieved to 10 mm. Commercial peat (SP) (*Sphagnum* moss) without previous fertilisation was used as diluent in the mixtures. PF and SP showed an acidic pH (5.1 for PF and 4.2 for SP), and low electrical conductivity (EC) values (1.12 dS m^-1^ for PF and 0.12 dS m^-1^ for SP), while GC had alkaline pH (8.3) and a high EC level (3.41 dS m^-1^). All the materials had the expected organic matter [30] contents (470 g kg^-1^ for GC, 820 g kg^-1^ for PF and 936 g kg^-1^ for SP) and total Kjeldahl N contents (28.5 g kg^-1^ for GC, 19.5 g kg^-1^ for PF and 9.3 g kg^-1^ for SP), as well as absence of phytotoxicity, with values of the germination index (69.4% for GC, 78.5% for PF and 84.8% for SP) higher than 60% according to Zucconi et al. [31].

### Formulation of growing media

The design of the formulations of the growing media was based on the triangular surface response method, in order to optimise the properties of the mixtures prepared with the different components. This method considers all the factors that influence the final properties of a mixture of components that must sum to a constant. In the case of a plant growing medium the sum of the proportions of each component reaches 100% [13]. In the mixture design of this experiment, the independent factors were the different proportions of the three components used in the preparation of the mixtures (GC, PF and SP). The standard mixture design used was the *Simplex-centroid* design [32], where the design points correspond to all permutations of the pure blends, of the binary blends and so on, depending on the number of components. This design was also augmented with three interior points. In this arrangement of design points, equally spaced proportions were tested for each factor in the model and all combinations of factor levels were tested. Ultimately, a total of ten growing media were elaborated by mixing GC and PF with peat in the proportions showed in Table 1, using pure peat (SP) as control treatment. Commercial perlite (Agrilit 1- Perlite Italiana) was added as inert substance at the proportion of 10% (v:v) to all the substrates, which were also fertilised at the beginning of the experiment with 5 g kg^-1^ of rock phosphate and 3 g kg^-1^ of potassium sulphate, both allowed in organic farming cultivation.

**Table 1:** Proportions (% by volume) of each component in the designed growing media.

Media	GC	PF	SP	Perlite
GC90%	90	0	0	10
PF90%	0	90	0	10
SP90%	0	0	90	10
GC-PF45%	45	45	0	10
GC-SP45%	45	0	45	10
SP-PF45%	0	45	45	10
GC60%	60	15	15	10
PF60%	15	60	15	10
SP60%	15	15	60	10
GC-SP-PF30%	30	30	30	10

GC: green compost, PF: palm fiber trunk wastes, SP: peat.

### Vegetal material and sowing

The experiment was carried out at an unheated polyethylene-covered greenhouse with natural daylight conditions at the IAMB-CIHEAM. Three vegetable species (melon, *Cucumis melo* L. ‘Carosello scopatizzo barese’); tomato (*Lycopersicon esculentum* Mill. ‘Rio Grande’); and lettuce (*Lactuca sativa* L. ‘Bionda Ortolani’) were selected and grown in foamed polystyrene plug trays with 24 cells of 55 mL, one seed being sowed per cell.

### Experimental design

The treatments (growing media) of this experiment were established in a completely randomised plot design with three replicates per treatment (one tray per replication). The treatments were irrigated daily or twice a day according to the environmental conditions by hose with mist nozzle, using enough water to avoid stress in the cultivated seedlings. Also, an organic liquid fertiliser made from yeast extract and brown sea algae *Ecklonia maxima* (Osbeck) Papenfuss (Algaren twin—Green HAS Italia s.p.a.—pH: 5.5; EC: 2.0 dS m^-1^; C: 14%; N: 2%) was applied once at the same rate for all the treatments at 30 days after sowing for tomato, at 25 days for melon, and at 20 days for lettuce.

### Variables determined in the seedlings

When the seedlings reached the commercial transplanting size, at 60 days for melon and tomato and at 40 days for lettuce, 15 seedlings were harvested at random from each experimental unit (a single plug tray), avoiding those placed next to the edges. In the stems, seedling length was measured from the root collar to the tip of the shoot (H); seedling diameter (D) was measured at the cotyledons node for lettuce and below the cotyledon node for melon and tomato.

In the leaves, seedling leaf area was measured using a leaf area meter. Specific Leaf area index (SLA), which is also used as transplant stress resistance index [14], was calculated as a ratio of seedling leaves area (cm^2^) to its dry weight (g). The number of true leaves (NL) of 5 sampled seedlings per each experimental unit was also counted at the end of the experiment. Foliar chlorophyll contents (SPAD values) were measured on 5 sampled leaves per each experimental unit by a chlorophyll meter (SPAD-502, Soil-Plant Analysis Development, Konica Minolta sensing, inc., Japan). Also, fresh weight (FW) of the total shoot (leaves and stem) was determined. Finally, the seedlings were dried (at 105°C in an air-forced oven for 24 h) to determine the dry weight (DW).

### Physico-chemical and chemical characteristics of growing media

The physico-chemical and chemical parameters of the raw materials were determined according to the methods described by Ceglie et al. [17]. In the growing media, pH, EC and bulk density were determined according to the standard European norms described by Tittarelli et al. [16].Water holding capacity of the growing media under different negative pressures was analysed in a sandbox (Eijkelkamp Giesbeek, The Netherlands). Water retention of the growth substrates was calculated at different negative pressures (pF0, pF1, pF1.7 and pF2), further elaboration was made according to De Boodt and Verdonck [33].

### Statistical methods

The mixture design models the synergistic and antagonistic effects of mixture components on a response variable, through the use of different equations. Suitable models for mixture designs consisting of three components include linear, quadratic and special cubic models [13]. In this study, for each seedling parameter, the significance of different models was tested. The models for the three variables case were:

1. Linear: y = b_1_x_1_+b_2_x_2_+b_3_x_3_;
2. Quadratic: y = b_1_x_1_+b_2_x_2_+b_3_x_3_ +b_12_x_1_x_2_+b_13_x_1_x_3_+b_23_x_2_x_3_
3. Special cubic: y = b_1_x_1_+b_2_x_2_+b_3_x_3_+b_12_x_1_x_2_+b_13_x_1_x_3_+b_23_x_2_x_3_+b_123_x_1_x_2_x_3_

where y is the dependent variable, b_i_ denote the regression coefficients (calculated from experimental data), and x_i_ are the independent variables. Each parameter was presented by the model which further contributes to explain the variability. The most significant model was used to plot the surface response on the ternary chart for each parameter. In addition, a mathematic function called desirability, which establishes the relationship between predicted responses on a dependant variable and the desirability of responses [34], was implemented taking into account the overall set of aforementioned parameters. A separate criterion per each parameter can be defined, establishing at which values should be either maximised or minimised or comprised into a specific range. On the basis of those criteria, the desirability function aggregated the obtained response areas in one multi response prediction, which ranges between 0.001 and 0.999 level (D_PL_). The D_PL_ values have been represented as a new response surface on a ternary chart that displays the overlapping of the single effect areas. This resulting response individuates the substrate formulations that best maintain optimal transplants characteristics, the values of the characteristics considered as optimal being based on commercial aspects and on the data reported in scientific literature. The colours of the surface were chosen to represent the optimal range of values. The qualitative scale green-yellow-red colour represents the range from the best to the worst values for each parameter. The area with the same colour indicates the range of values that corresponds to the same response characteristics.

The mixture design based on the triangular surface response method and the desirability function were defined and analysed by the design of experiment (DOE) Design-Ease software v.9 (Stat-Ease Inc., 2014). Mean values of the seedlings parameters were statistically analysed by ANOVA to evaluate the significant effect of the growing media factor. Then, the Tukey post-hoc test was used to evaluate differences between groups of mean per each treatments, using as data analysis software system StatSoft, Inc. STATISTICA (data analysis software system, version 10. www.statsoft.com).

## Results and Discussion

### Physico-chemical and physical properties of the growing media

The main physico-chemical and physical properties of the different growing media elaborated compared to the values established for an ‘ideal’ substrate [35, 6] are shown in Table 2. The incorporation of GC produced a clear increase in the pH values and in the salinity contents of the growing media. In particular, the mixtures with the greatest proportions of GC showed the highest pH and EC values, these values being higher than those suggested as optimum values (pH in the range 5.3–6.5 and EC < 0.5 dS m^-1^) [35, 6]. This fact was also reported by other authors in experiments of peat substitution using composts with either similar [16, 17] or different origin [11, 22, 26] from that used in this study. Regarding the physical properties, all the mixtures showed suitable values of the bulk density (< 0.4 g cm^-3^), observing in the substrates with higher percentages of PF (PF90% and PF60%) the lowest bulk density values. Bulk density and the total pore space (TPS) are parameters inversely correlated in the growing media. Therefore, low bulk density is associated to high free pore space, which could potentially favour plant root growth. This fact was in accordance with the values of the TPS obtained for the different substrates. The mixtures with the higher proportion of SP and PF showed the significantly greatest values of TPS. These values resulted slightly under the ‘ideal’ substrate threshold. While air volume (AV) values were quite lower than the limit range suggested as optimum (20–30% vol). Usually, this AV percentages might be a problems for plant growth, especially in plug trays with small container, due to the poor drainage after watering [23]. It wasn’t the case due to the fact that other limiting parameters, such as the total available water (TAW) and easily available water (EAW) were, in general, within the recommended ranges [35, 6]. The best values of TAW and EAW were observed in the substrates with higher proportion of PF and lower proportion of GC (PF90%, PF60% and SP-PF45%), even showing higher values than the substrates with greater percentage of peat (SP90% and SP60%). This fact could imply a suitable retention of water, which avoids frequent leaching during irrigation. The low values of the air volume and the high values of the TAW and EAW are probably consequence of the small particle size of the materials (GC and PF) used, which were previously sieved to 10 mm. Noguera et al. [6] reported in a study on the physical properties of different coconut coir dust samples that both easily available water and total water holding capacity diminished proportionally with increasing coarseness index, while the air content was positively correlated.

**Table 2:** Physico-chemical and physical properties of the growing media.

Substrate	pH	EC(dS m^-1^)	BD(g cm^-3^)	TPS(% vol)	AV(% vol)	TAW(% vol)	EAW(% vol)	LAW(% vol)	WBC(% vol)
Optimum range^1^	5.3–6.5	≤ 0.5	≤ 0.4	> 85	20–30	24–40	20–30	—	4–10
GC90%	7.8a	3.35a	0.33a	67.1c	3.23bc	20.3cd	19.2bc	43.5bc	1.17c
PF90%	5.6e	0.67f	0.07g	75.8abc	4.72bc	35.4a	33.8a	35.6bc	1.57c
SP90%	4.2h	0.17h	0.13f	81.9a	5.93ab	35.0a	19.9bc	41.0bc	15.1a
GC-PF45%	7.0b	2.96b	0.19cd	68.8bc	10.66a	21.4bcd	14.6c	36.7bc	6.80bc
GC-SP45%	6.1d	2.51c	0.21c	73.8abc	2.61c	16.1d	9.30c	55.1a	6.84bc
SP-PF45%	4.8g	0.42g	0.13f	77.4ab	6.68ab	29.9abc	19.0bc	40.8bc	10.9ab
GC60%	6.9b	3.04b	0.25b	69.9bc	4.42bc	19.9cd	10.5c	45.6ab	9.42ab
PF60%	6.6c	1.82d	0.12f	72.4bc	6.49ab	31.9ab	30.6ab	34.0c	1.30c
SP60%	5.1f	1.35e	0.15ef	73.9abc	5.51bc	25.0abcd	18.4bc	43.4bc	6.60bc
GC-SP-PF30%	6.2d	3.03b	0.17de	72.9abc	5.23bc	31.9ab	30.8ab	35.8bc	1.11c

GC: green compost, PF: palm fibre trunk wastes, SP: peat.

^1^According to Abad et al. [35] and Noguera et al. [6].

EC: electrical conductivity; BD: bulk density; TPS: total pore space; AV: air volume; TAW: total available water; EAW: easily available water; LAW: less available water; WBC: water buffering capacity.

Mean values (n = 4) in columns followed by the same letter are not statistically different according to the Tukey test at P < 0.05.

### Response surface analysis to evaluate the effect of each component on substrate properties

Based on the data obtained from the experiment, empirical models to describe the interrelationship between dependent and independent variables by equations were developed. The independent variables used and their variation limits were: GC proportion (from 0% to 90% v/v); PF proportion (from 0% to 90% v/v) and SP proportion (from 0% to 90% v/v). The dependant variables were all the physico-chemical and physical properties determined in the substrates elaborated: pH, EC, BD, TPS, AV, TAW, EAW, less available water (LAW) and water buffer capacity (WBC); and the parameters determined in the seedlings (H, D, SI, NL, FW, SPAD and DW for tomato and melon; H, NL, LA, LAI, FW, SPAD and DW for lettuce). Table 3 shows the models used to fit each parameter studied to the proportions of each component in the mixture, together with their statistical significance. Also, the equations obtained with the corresponding statistical parameters (R^2^ and predicted R^2^) to measure the correlation among the real variables and the correlation among the predicted variables, respectively, have been included. All the models showed good significance (P value < 0.05) and, in general, suitable values of the real correlation (R^2^) and predicted correlation (Predicted R^2^), allowing an accurate description of most of the parameters studied. In an experiment of peat substitution with municipal solid waste compost and composted pine bark, Moldes et al. [13] also reported models with a good correlation and significance to fit experimental data related to plant parameters in different crop species to the proportion of each component considered in the mixtures studied.

**Table 3:** Models obtained with the significance level and statistical parameters for the determined parameters in the growing media and in the seedlings of tomato, melon and lettuce.

Parameter	Model	Pvalue	Equation	R^2^	Pred. R^2^
*Substrate* pH	Linear	< 0.0001	pH = 7.94GC + 5.94PF + 4.21SP	0.9433	0.8704
*Substrate* EC	Linear	0.0013	(EC)^2^ = 13.05GC + 1.85PF + 0.25SP	0.8499	0.6959
*Substrate* BD	Quadratic	< 0.0001	BD = 0.34GC + 0.075PF + 0.13SP—0.056GC·PF—0.096GC·SP + 0.099PF·SP	0.9979	0.9625
*Substrate* TPS	Linear	0.0165	TPS = 70.17GC + 80.55PF + 67.05SP	0.6904	0.2357
*Substrate* AV	Quadratic	0.0010	(AC)^2^ = 12.34GC + 1.46PF + 65.84SP + 214.2GC·PF—4.5GC-113.4PF·SP	0.9850	0.7178
*Substrate* AW	Special cubic	0.0062	(AW)^-1.5^ = -0.014GC + 0.011PF + 0.033SP—3.98·10^-3^GC·PF + 4.23GC·SP + 1.51PF·SP—17.01GC·PF·SP	0.9873	0.5374
*Substrate* EAW	Special cubic	0.0153	(EAW)^-0.5^ = 0.35GC + 0.16PF + 0.16SP + 0.46GC·PF + 13.44GC·SP + 4.38PF·SP—51.97GC·PF·SP	0.9765	0.2519
Tomato-H	Linear	0.0169	(H)^2^ = 0.0392GC + 6.657·10^-3^PF—6.575·10^-4^SP	0.7435	0.5172
Tomato-NL	Quadratic	0.0022	NL = -0.16GC + 3.68PF + 3.73SP + 11.74GC·PF + 7.20GC·SP + 1.44PF·SP	0.9991	0.9499
Tomato-D	Quadratic	0.0144	D = -1.11·10^-3^GC + 3.60·10^-3^PF + 3.52·10^-3^SP + 1.04·10^-4^GC·PF + 1.16·10^-4^GC·SP + 2.96·10^-5^PF·SP	0.9733	0.6052
Tomato-FW	Quadratic	0.0045	1/FW = 2.62GC + 0.57PF + 0.76SP—4.25GC·PF—4.66GC·SP—1.09PF·SP	0.9879	0.7729
Tomato-DW	Quadratic	0.0008	1/DW = 28.11GC + 4.24PF + 6.40SP -38.59GC·PF—45.12GC·SP—10.45PF·SP	0.9961	0.9355
Tomato-SPAD	Quadratic	0.0040	1/SPAD = 0,053GC + 0.078PF + 0.039SP—0.149GC·PF—0.036GC·SP—0.088PF·SP	0.9889	0.7782
Melon-H	Quadratic	0.0061	(H)^-2^ = 0.034GC + 0.016PF + 0.054SP—0.067GC·PF—0.077GC·SP—0.083PF·SP	0.9851	0.7772
Melon-NL	Quadratic	0.0015	(NL)^-3^ = 0.17GC + 0.043PF + 0.099SP—0.24GC·PF—0.32GC·SP—0.098PF·SP	0.9814	0.6299
Melon-FW	Special cubic	0.0083	(FW)^-3^ = 0.20GC + 0.029PF—0.09SP—0.41GC·PF + 0.26GC·SP + 0.39PF·SP—1.67GC·PF·SP	0.9972	0.8261
Melon-DW	Linear	0.0219	1/DW = 4.49GC + 1.57PF + 5.90SP	0.7203	0.3587
Melon-SPAD	Quadratic	0.0038	1/(SPAD+15) = 0.025GC +0.045PF + 0.030SP—0.043GC·PF + 6.83·10^-3^GC·SP—0.051PF·SP	0.9701	0.7160
Lettuce-NL	Linear	0.0377	(L)^-2^ = 0.047GC + 1.09PF + 0.026SP	0.6646	0.1330
Lettuce-FW	Quadratic	0.0021	(FW)-3 = 5.23GC + 2.15PF + 1.81SP—12.84GC·PF—8.16GC·SP—7.78PF·SP	0.9778	0.5669
Lettuce-DW	Quadratic	0.0009	(L)^-3^ = 5565GC + 783PF + 1496SP—11498GC·PF—10599GC·SP—3731PF·SP	0.9855	0.7429
Lettuce-SPAD	Linear	0.0248	SPAD = 28.2GC + 19.3PF + 29.5SP	0.7084	0.2224
Lettuce-LAI	Linear	0.0065	(LAI)^-1^ = 2.71·10^-3^GC + 5.12·10^-3^PF + 2.98·10^-3^SP	0.7629	0.4546

H: total length; D: stem diameter; SPAD: foliar chlorophyll values; NL: leaves number; LA: leaves area; LAI: leaf area index; FW: fresh weight of seedlings; DW: dry weight of seedlings. For other abbreviations, see Table 2.

R^2^: R-squared; Pred. R^2^: predicted R-squared.

**Fig 1:**
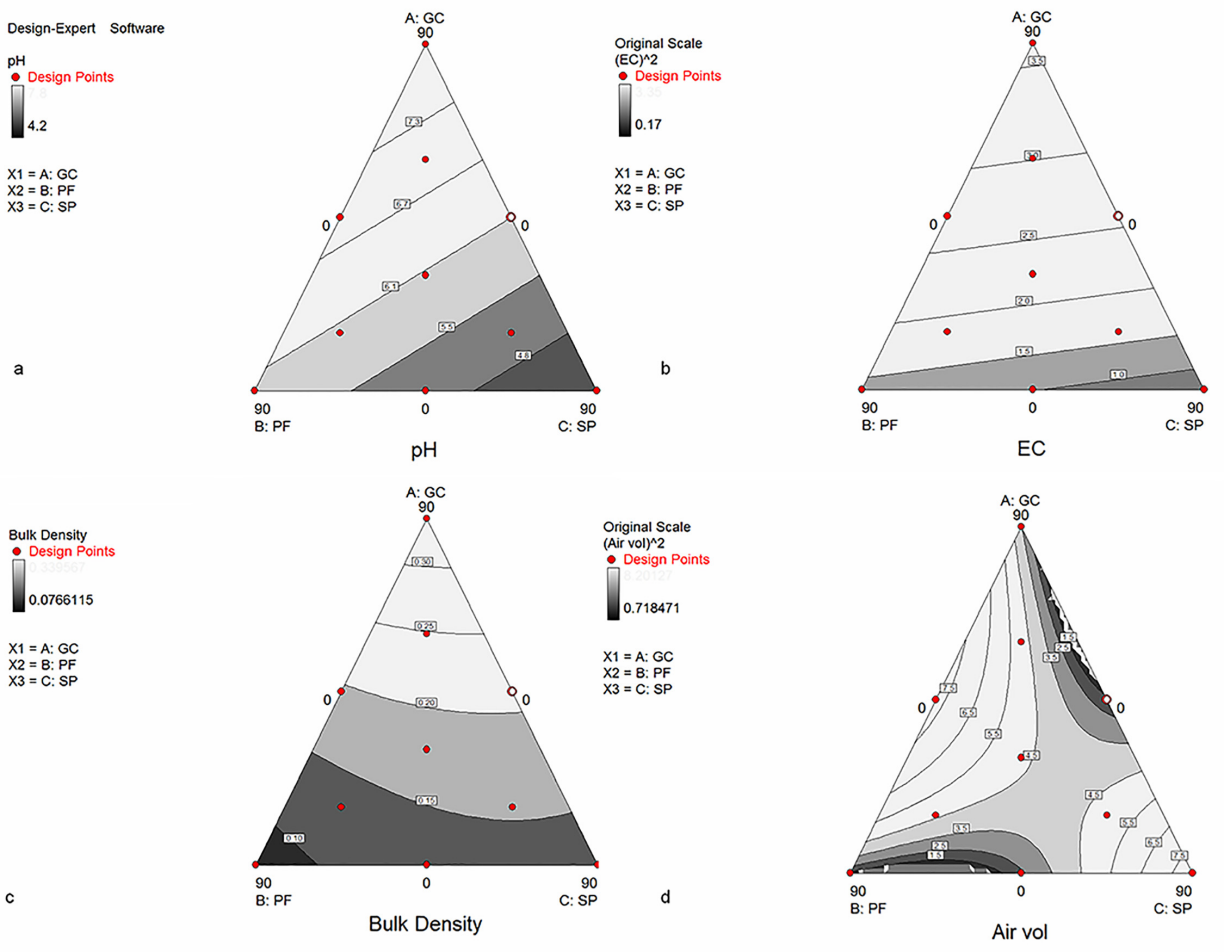
Triangular surface response for several physico-chemical (a and b) and physical (c and d) parameters in the substrates elaborated. (a) pH. (b) EC: electrical conductivity. (c) bulk density. (d) air volume.

Fig 1 report the triangular surface response of several physico-chemical and physical parameters of the growing media, according to the models previously commented (Table 3). The physico-chemical parameters (pH and EC) fitted to a linear model, while the physical parameters BD and AV fitted to a quadratic one. In case of pH (Fig 1A), the model predicts that the highest pH values are obtained with mixtures with GC as main component and SP in the minimal proportion. This fact is in accordance with the increase of pH with the increasing percentage of compost reported in other experiments of peat substitution using compost [16]. Concerning the salinity (Fig 1B), to obtain growing media with EC values lower than 2 dS m^-1^ using these ingredients, the proportions of the component GC must be lower than 30% v/v, being the mixtures with the highest percentage of SP in the mixture within the area corresponding to the lowest EC values.

On the contrary, the physical properties studied (BD and AV) showed a different behaviour (Fig 1C and 1D, respectively). The lowest values of BD are in the area where the mixtures must be mainly composed by PF and SP, this fact confirms the results observed and previously commented, which showed that the mixtures with the greatest percentages of PF had the lowest BD. On the other hand, the air volume (AV) displayed a more complex behaviour. The highest values of this parameter, desirable for a good plant development, especially for seedling production in small containers [23], are obtained in two different areas, one corresponding to mixtures with the highest proportion of peat (90% v/v) and the other, corresponding to mixtures only with GC and PF in the same proportion (45% v/v).

### Plant response with the different growing media: comparative effects and use of the desirability functions

The effects of the substrates elaborated on the morphological parameters and aerial biomass production for tomato, melon and lettuce seedlings are shown in Table 4.

**Table 4:** Comparative effects of the different growing media used on the morphological parameters and aerial biomass production for melon, tomato and lettuce seedlings.

	GC90%	PF90%	SP90%	GC-PF45%	GC-SP45%	SP-PF45%	GC60%	PF60%	SP60%	GC-SP-PF30%
*Tomato*										
H (cm)	3.29g	5.13bcd	6.97ef	7.80de	8.11cde	9.54bc	9.91fg	11.1a	13.4a	13.4a
D (cm)	0.17d	0.25bcd	0.32bc	0.32bc	0.32bc	0.34bc	0.38cd	0.39a	0.40ab	0.42ab
SPAD	12.7b	25.7ab	28.3ab	27.3ab	35.0a	27.3ab	27.6ab	32.0a	31.3ab	35.0a
NL	2.1e	3.2cd	3.6bcd	3.6bcd	3.7cde	3.7abcd	4.0d	4.3a	4.6abc	4.7ab
FW (g)	0.64e	1.76cd	1.29cde	2.00bc	1.97bc	2.67b	1.04de	3.93a	4.08a	4.28a
DW (g)	0.05e	0.23c	0.15cde	0.15cde	0.17cde	0.37b	0.09de	0.52a	0.48a	0.35b
*Melon*										
H (cm)	1.84e	7.86c	4.29d	11.2b	6.36cd	8.08c	7.13c	11.8ab	13.3a	12.9ab
D (cm)	0.36cd	0.40bc	0.29d	0.48ab	0.35cd	0.40bcd	0.35cd	0.52a	0.50a	0.50a
SPAD	20.4ab	19.1ab	24.6a	24.1a	23.8a	18.6ab	22.2a	24.8a	30.3a	25.7a
NL	1.8d	2.9a	2.1c	2.8ab	2.6bc	2.8ab	2.3bc	2.9a	2.9a	2.9a
FW (g)	1.71de	3.21b	1.12de	4.34a	2.04cd	2.46bc	2.18c	4.75a	4.95a	4.95a
DW (g)	0.17d	0.43abc	0.11d	0.38abc	0.19cd	0.24bcd	0.32abc	0.48a	0.49a	0.49a
*Lettuce*										
H (cm)	2.48e	3.52de	3.93de	3.91de	4.27cd	5.67bc	3.79de	7.17bc	10.4a	6.02b
NL	3.1e	5.7cd	5.2d	4.2de	5.4cd	8.2ab	4.6de	8.0ab	9.7a	7.2bc
LA (cm^2^)	20.5d	18.6d	25.3d	32.8d	28.6d	72.6c	23.5d	94.2b	160a	70.5c
LAI (cm^2^ g^-1^)	280abc	178c	357a	195bc	325ab	261abc	268abc	264abc	302abc	246abc
FW (g)	0.52e	0.76d	0.80d	2.56d	0.91d	2.60c	0.71d	3.57b	6.03a	2.56c
DW (g)	0.06d	0.11c	0.08c	0.11c	0.10c	0.29b	0.09c	0.36b	0.54a	0.30c

GC: green compost, PF: palm fibre trunk wastes, SP: peat.

H: total length; D: stem diameter; SPAD: values of foliar chlorophyll contents; NL: number of leaves; LA: area of leaves; LAI: leaf area index; FW: fresh weight of seedlings aerial part; DW: dry weight of seedlings aerial part.

Mean values (n = 15) in rows followed by the same letter are not statistically different according to the Tukey test at P < 0.05.

**Fig 2:**
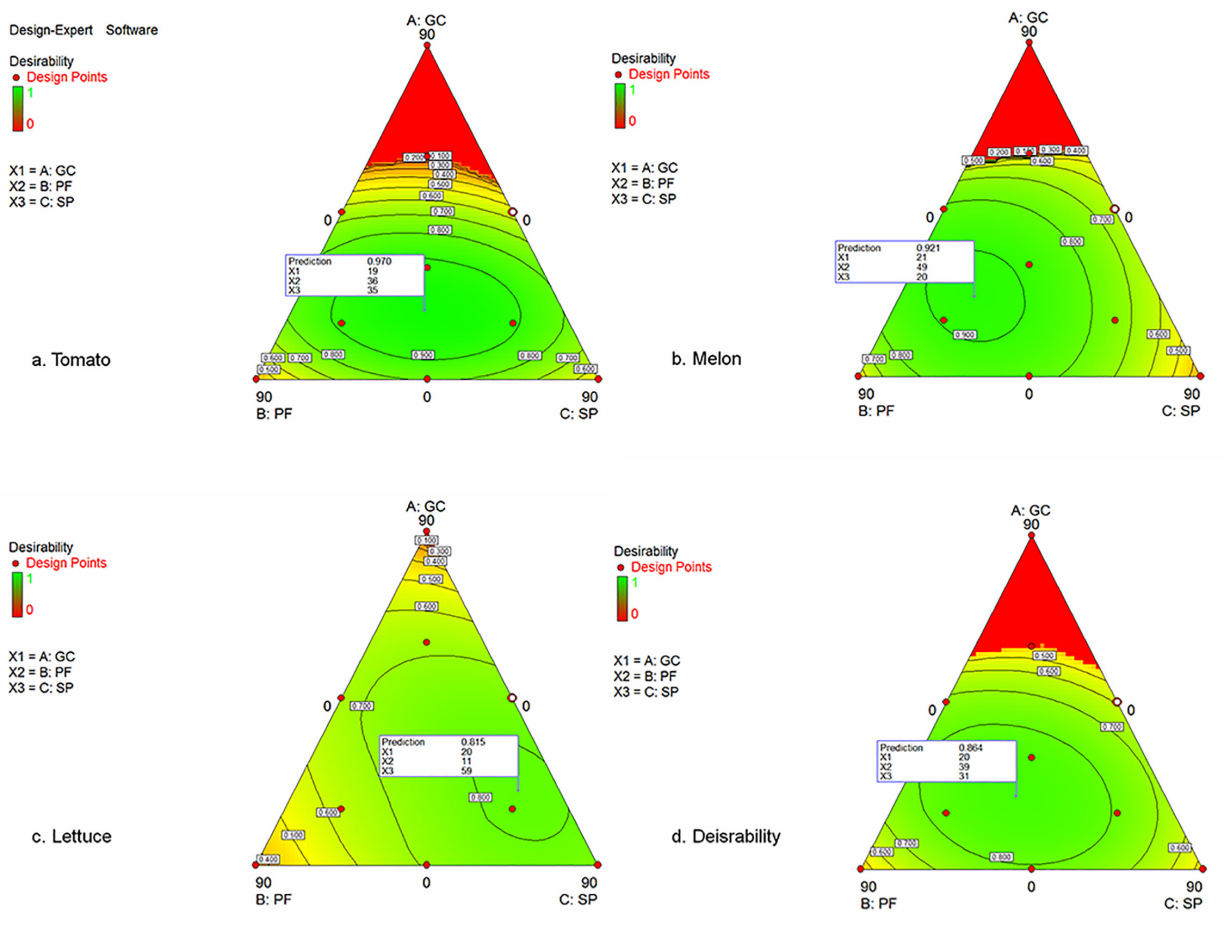
Desirability functions considering the substrates elaborated for the vegetal species (a—c) and for all the vegetal species studied (d). The flag reports the highest prediction level of the optimal transplants performance and the corresponding proportions of each component. Green colour in the surface represents the area in which the optimal transplants characteristics are obtained. (a) Tomato. (b) Melon. (c) Lettuce. (d) All the vegetal species studied.

In addition, Fig 2 shows the desirability functions for each type of vegetal species and for all these species grouped. Significant differences can be observed for the morphological and yield parameters for the different growing media elaborated (Table 4). The effect on these parameters of the growing media were similar in the tomato and melon seedlings, obtaining, in general, the highest values of these parameters for the mixtures SP60%, PF60% and CG-SP-PF30%. In the lettuce seedlings, the behaviour of the parameters was slightly different to that observed in tomato and melon, obtaining the highest values of the morphological parameters H, NL and LA, as well as for the yield parameters FW and DW, only with the mixture SP60%. On the other hand, the parameter LAI, only studied in the lettuce seedlings, showed a different behaviour, obtaining the greatest values (not significant difference from SP60%) with the mixture SP90% (Table 4). This different performance was also reflected in the desirability functions of each plant species, observing a similar trend in the seedlings of melon and tomato, quite different from that observed for the lettuce seedlings (Fig 2). The desirability functions establish the relationship between predicted responses on a dependant variable and the desirability of responses, using a set of parameters. In the case of the tomato crop, the desirability function considered the following parameters: H, NL, FW, DW, D and SPAD, which were the parameters that, previously, fitted best the models. The criteria with which these parameters were set, was to maximize their values. Thus, the desirability function for the tomato seedlings (Fig 2A) showed that the optimal transplant parameters (those previously commented) would be obtained, with a prediction level D_PL_ = 0.970 (97%), with the mixture in the proportions of 19%GC:36%PF:35%SP. For the melon seedlings, the parameters selected were the same as for the tomato seedlings, considering as criterion in all of them the maximum value. In this case, the best response for these parameters (Fig 2B) would be obtained at a D_PL_ = 92%, with a mixture composed by 21%GC:49%PF:20%SP. However, for the lettuce seedlings, the parameters selected were NL, LAI, SPAD, FW and DW, also considering as criterion for all the parameters the maximum value, and the desirability function obtained was quite different. In this crop, the best result would be obtained at a lower maximum prediction level (D_PL_ = 81%) and for a mixture with a higher proportion of peat (20%GC:11%PF:59%SP). All these aspects can be summarized in the desirability function obtained by analysing all the plant species studied (tomato, melon and lettuce), at the same time. All the parameters considered in the previous desirability functions have been included, also with the same criteria. The prediction of this function shows the maximum value (D_PL_ = 86%) with a mixture based on 20%GC:39%PF:31%SP. Furthermore, an optimal desirability level higher than 80% may be obtained in the homogeneous surface around the maximum point, as it is shown in Fig 2D. In the ternary area, each proportion of components in the mixture that varies in the same range of DPL results in similar transplants performances, allowing the choice of the most feasible component percentage in this range for the optimal substrate design. In this experiment, the results predicted by models were in agreement with the measured ones and with those observed in other studies of peat substitution using similar materials [16, 18], confirming the significant capacity of the desirability functions to describe and predict the data obtained.

## Conclusions

In general, the results obtained in this experiment have shown that green waste compost and palm fibre waste derived substrates were suitable for their use in seedling production, obtaining the best results in all the plant species studied with the mixture 20%GC:39%PF:31%SP and with the potential mixtures included in the near surface. Moreover, this result overlaps with that predicted using the mixture design and surface response methodology. The desirability functions of the plant species also reflected the different responses found in the plant parameters studied, observing a similar trend in the seedlings of melon and tomato which achieve the optimal performances with 20% of peat (for tomato) and 35% (for melon) in the growing media. While lettuce seedlings require as much as 60% peat to reach its peak of desirability (86%). The results obtained so far are very encouraging and the potential fields of application of this methodology in nursery activities are numerous. From the technical point of view, keeping the three organic materials utilized in our research, it would be possible to maximise a desirability function for any vegetable crop. The methodology applied in this manuscript can be used to maintain a desirability level >80% for all vegetable seedlings sold by a nursery. The study of the desirability function of any innovative organic component would improve its use efficiency in growing media formulation. This would ensure large margins of profitability for substrates producers and for importers/exporters of substrate components. From the normative point of view, this methodology would allow policy makers to better set limits in peat substitution either for organic and conventional nursery activity. Therefore, this methodology constitutes an innovative and useful approach with a significant capacity to describe and predict the data obtained in peat substitution experiments. It simplifies the decision-making process to identify the mixture and to obtain ‘tailored’ substrates with optimal seedling response in terms of plant growth and productivity. Finally, further researches should be carried out to confirm the results obtained and to transfer this methodology at the nursery companies level.
